# Measurement of apoptosis by SCAN^©^, a system for counting and analysis of fluorescently labelled nuclei

**DOI:** 10.15698/mic2014.12.180

**Published:** 2014-11-26

**Authors:** Neta Shlezinger, Elad Eizner, Stas Dubinchik, Anna Minz-Dub, Rachel Tetroashvili, Adi Reider, Amir Sharon

**Affiliations:** 1 Department of Molecular Biology and Ecology of Plants, Faculty of Life Sciences, Tel Aviv University, Tel Aviv 69978, Israel.; 2 Department of Physical Electronics, Fleischman Faculty of Engineering, Tel Aviv University, Tel Aviv 69978, Israel.

**Keywords:** apoptosis, PCD, fungi, DNA condensation, TUNEL

## Abstract

Apoptosis-like programmed cell death (A-PCD) is a universal process common to all types of eukaryotic organisms. Because A-PCD-associated processes are conserved, it is possible to define A-PCD by a standard set of markers. Many of the popular methods to measure A-PCD make use of fluorescent ligands that change in intensity or cellular localization during A-PCD. In single cell organisms, it is possible to quantify levels of A-PCD by scoring the number of apoptotic cells using flow cytometry instruments. In a multicellular organism, quantification of A-PCD is more problematic due to the complex nature of the tissue. The situation is further complicated in filamentous fungi, in which nuclei are divided between compartments, each containing a number of nuclei, which can also migrate between the compartments. We developed SCAN^©^, a System for Counting and Analysis of Nuclei, and used it to measure A-PCD according to two markers - chromatin condensation and DNA strand breaks. The package includes three modules designed for counting the number of nuclei in multi-nucleated domains, scoring the relative number of nuclei with condensed chromatin, and calculating the relative number of nuclei with DNA strand breaks. The method provides equal or better results compared with manual counting, the analysis is fast and can be applied on large data sets. While we demonstrated the utility of the software for measurement of A-PCD in fungi, the method is readily adopted for measurement of A-PCD in other types of multicellular specimens.

## INTRODUCTION

Apoptosis was initially described in animals, where it plays central roles in development and homeostasis 1,2. Activation of apoptosis, by external or internal stimuli, drives a set of coordinated intracellular processes, which terminate with the formation of apoptotic bodies and cell death. The most significant processes that define apoptosis include ions and protein leakage from the mitochondria, phosphatidylserine externalization from the inner to the outer cell membrane, chromatin condensation, DNA fragmentation, membrane blabbing, increased caspase activity and appearance of apoptotic bodies 3. Following the discovery of apoptosis in animals, programmed cell death with similar characteristics was described in additional systems, including single-celled organisms 4,5. The similarities in the sequence of events in animals and low eukaryotes promoted the use of simple systems to study apoptosis and apoptosis-related processes. In particular, *Saccharomyces cerevisiae *has become a model system for evaluation of human apoptotic genes and apoptosis-related processes such as autophagy and diseases 6,7,8. Additionally, fungi are used to study the role of apoptosis and apoptosis-like programmed cell death (A-PCD) in aging, pathogenicity and stress responses 9,10.

Because apoptosis-associated processes are conserved, it is possible to identify apoptosis and A-PCD by a universal set of assays. A wide range of methods have been developed for this purpose, which measure cytological, molecular and biochemical parameters that are typical of apoptotic cells 11,12,13,14,15. In this respect, it is important to distinguish between qualitative methods, such as detection of membrane blabbing by electron microscopy, or detection of DNA fragmentation by appearance of DNA laddering, and methods that allow quantitative measures, which are more relevant in comparative studies, e.g., of mutants, treatments, etc. In many of the popular methods, fluorescent ligands are used to label cells, nuclei or specific proteins, and apoptosis is evaluated according to changes in the fluorescent signal. For example, phosphatidylserine externalization can be detected by reaction with fluorescently-labelled annexin V, DNA strand breaks are detected by adding a fluorescent tag to the exposed ends of the DNA using terminal dUTP nick end-labeling (TUNEL), and DNA fragmentation and chromatin condensation can be detected by staining with DNA-specific fluorescent dyes, such as 4',6-diamidino-2-phenylindole (DAPI) or Hoechst 33342 16,17. The final stages of apoptosis, which are usually executed by caspases, can be detected by accumulation of a fluorescent product from a non-fluorescent caspase substrate.

In single-celled organisms, such as yeasts and single cell algae, the level and distribution of the fluorescent signal can be used to quantify A-PCD 18,19. In some cases high throughput measurements are possible with the aid of flow cytometry 20,21,22. Relative levels of apoptosis can also be estimated by measuring fluorescence intensity, e.g., following caspase activity assay 23. While widely accepted, these methods have several limitations. Some of the methods require the use of expensive reagents and extensive processing, which limit the number of samples that can be analyzed. For example, FITC-annexin V is an expensive reagent and the procedure can’t be used on samples with a cell wall. Hence, for organisms such as plants, algae or fungi, it is necessary to first digest the cell with lytic enzymes to produce protoplasts, a harsh process that induces stress in the tissue. Fluorescence activated flow cytometry (FACS) is often used to quantify A-PCD in processed samples, but this does not provide details on spatial or temporal changes of the fluorescent signal. Furthermore, the use of FACS is limited to the single-celled specimens.

Various methods and instruments can be used for visualization and analysis of A-PCD in multicellular samples. A simple way to obtain quantitative information is capturing images, using a fluorescent microscope and a digital camera, and then counting the apoptotic signals, either manually or with the aid of image analysis software 24,25. Other methods include the use of dedicated instruments that are equipped with appropriate optics and software. These systems can be used to simultaneously measure A-PCD in large number of samples and are the nearest equivalent of flow cytometry 26,27. While the instruments are rather efficient in analysis of uniform samples, such as animal cells, they are much less suitable for analyses of heterogeneous samples, such as plant tissues and fungal mycelium. In addition, the high cost of the instruments makes them less accessible, in particular to smaller labs.

Here we report on a system for analyses of A-PCD in fungal hyphae. The system is composed of several modules, which enable automatic quantification of nuclei with chromatin condensation and DNA strand break in large datasets according to nuclei-associated fluorescent markers. While we developed the system for measurement of A-PCD in fungi, it can be adopted with minor adjustments for measurement of A-PCD in other types of organisms and tissues.

## RESULTS AND DISCUSSION

The SCAN**^©^** (System for Counting and Analysis of Nuclei) software contains three separate modules: (i) Layers, for counting of nuclei in hyphae, (ii) Condensation, for counting and calculating the ratio of nuclei with condensed chromatin, and (iii) TUNEL, for measurement and calculation of the ratio of TUNEL stained nuclei. A graphic user interface (GUI) allows the user to choose the modules, determine parameters, and perform analyses either on single images or on a library of images (Fig. S1). In order to test the accuracy of the software, we analyzed sets of images by the different modules of the SCAN**^©^** software, and compared the results with manual counting of the same samples.

### The Layer module for counting nuclei in hyphae

**Figure 1:**
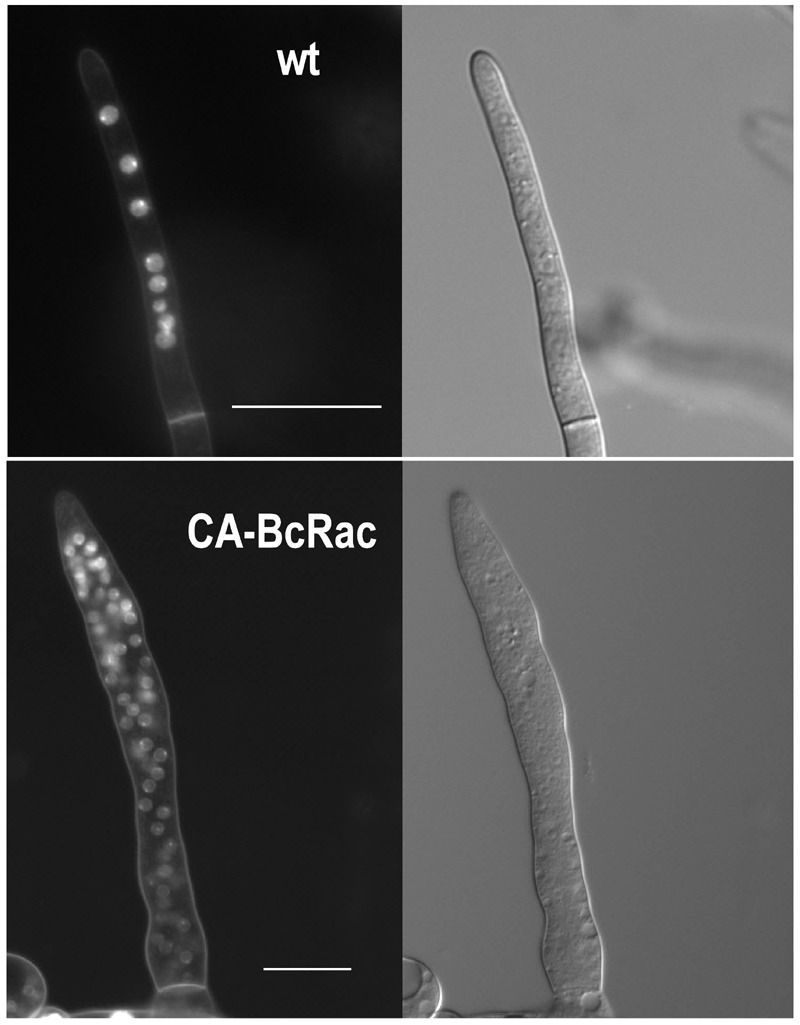
FIGURE 1: DAPI-stained nuclei in hyphae of wild type and CA-BcRAC *B. cinerea *strains. Wild type hyphae (top) contain several nuclei in each compartment. Hyphae of the CA-BcRAC (bottom) contain up to 100 nuclei in a single hypha compartment Scale bar = 20 µm.

**Figure 2:**
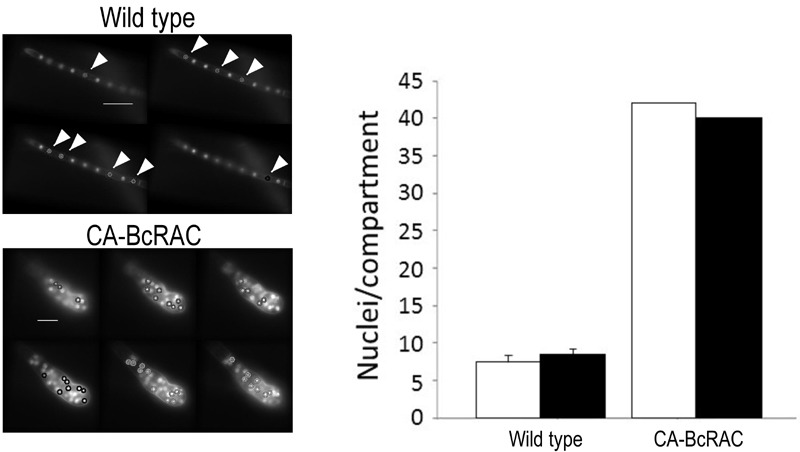
FIGURE 2: Nuclei counts in hyphae of wild type and CA-BcRAC strain. Hyphae were harvested, stained with Hoechst 33342 and visualized at 400x magnification. Images were captured using the z-stack function with 0.3 µm sections. Images show consecutive z sections of wild type (top) and CA-BcRAC (bottom) hyphae. In each section the most distinct nuclei are automatically marked by the software (also manually marked by arrows in images of the wild type hyphae). Scale bar = 20 µm. The number of nuclei between the hyphal tip and the first septum was counted manually (white bars) and by the Layer module of the SCAN**^©^** software (black bars). For the wild type, the graph shows the average number ± s.d. of nuclei counted in 30 hyphal segments. For CA-BcRAC graph shows the actual number of nuclei counted in a specific hyphal segment.

*B. cinerea* hyphae are multinucleated (Fig. 1). Because not all nuclei are visible in a single focal plane, it is often difficult to determine the exact number of nuclei from a single microscopic image. The way to overcome this problem is to count nuclei in a series of images taken at different focal planes (z stacks), which is laborious and time consuming. Counting of nuclei in hypernucleated mutants is especially difficult, because overlap of nuclei and distortion of the signal (Fig. 1). To overcome these problems, we developed the Layers module for unbiased, automated counting of all stained nuclei in a defined hyphal area. The procedure includes identification of the hyphal contours, detection of the sharpest images of the nuclei in each focal plane, so that each nucleus is counted only once, and then determination of a final number of nuclei in a defined hyphal compartment (Fig. 2). This procedure is fast and can be performed on a batch file of numerous samples, saving long hours of tedious work. To determine the accuracy of the software we compared software results with manual counts of nuclei in 30 samples. The average numbers were slightly higher for the manual counts (8.5 versus 7.5) however, the variance within counts was similar (Fig. 2). This result indicates that the software is more conservative than manual counts, but it has same level of reproducibility. Because in most cases the relative number of nuclei is more important than the exact number, the small difference in absolute number of nuclei is not expected to cause bias. The software was also proven highly efficient in counting nuclei in hypernucleated hyphae (Fig. 2). The number of nuclei obtained by manual counting was slightly higher than that provided by the software, however in this case the reliability of the manual counts might be lower due to overlapping signals, which are hard to decipher and might lead to scoring errors. Using the software in this case is not only easier and faster, but it also provides more accurate and reproducible results.

### Measurement of apoptotic nuclei

**Figure 3:**
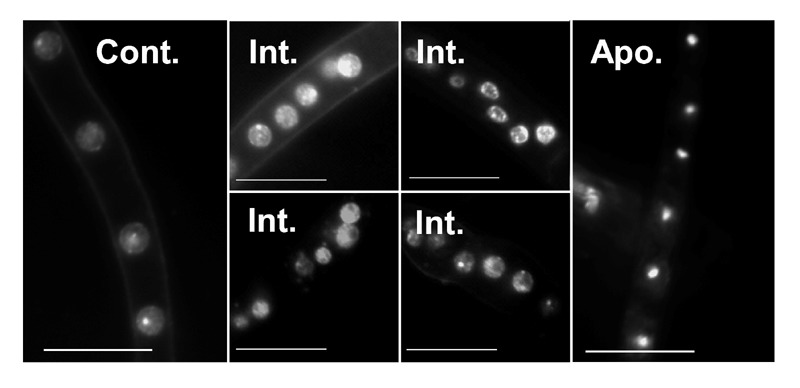
FIGURE 3: Changes in nuclei morphology in response to PCD-inducing conditions. Cultures were grown for 24 h in PDB, then H_2_O_2_ was added to a final concentration of 10 mM. The mycelia were harvested after 4 h of incubation with H_2_O_2_, processed and stained with Hoechst 33342. Samples were visualized at 1000x magnification. Control, time zero, cells are loosely and evenly stained, the nucleolus is clearly visible (arrow); Int., intermediate stages of apoptosis, accumulation of nuclear fragments is visible; Apoptotic, highly condensed nuclei represent the final stage of apoptosis. Scale bar = 10 µm.

Quantitative assessment of A-PCD levels in filamentous fungal species is challenging because of uneven progression of PCD in different parts of the colony, multi-nucleate nature of the hyphae and inability to count single units (i.e. single cells like in yeasts). Furthermore, certain methods are not readily applicable. For example, staining of externalized phosphatidylserine on the outer membrane with annexin V can only be applied after digestion of the cell wall, DNA ladder either doesn’t form or is very hard to detect in fungi, and caspase activity assays are either not specific enough or provide only qualitative results. Of all the classical markers associated with apoptosis, chromatin condensation and nuclei morphology remain the easiest markers to detect and score (Fig. 3). But even the use of these markers is not trivial, since they vary according to the apoptotic stage in individual hypha. In order to quantify A-PCD in filamentous fungi, it is necessary to count nuclei in a large number of samples, a tedious and biased-prone process. To solve this problem, we developed two modules of the SCAN**^©^** software: Condensation, for measurement of nuclei with condensed chromatin, and TUNEL, for measurement of nuclei with DNA strand breaks.

### The Condensation module for counting of condensed nuclei

**Figure 4:**
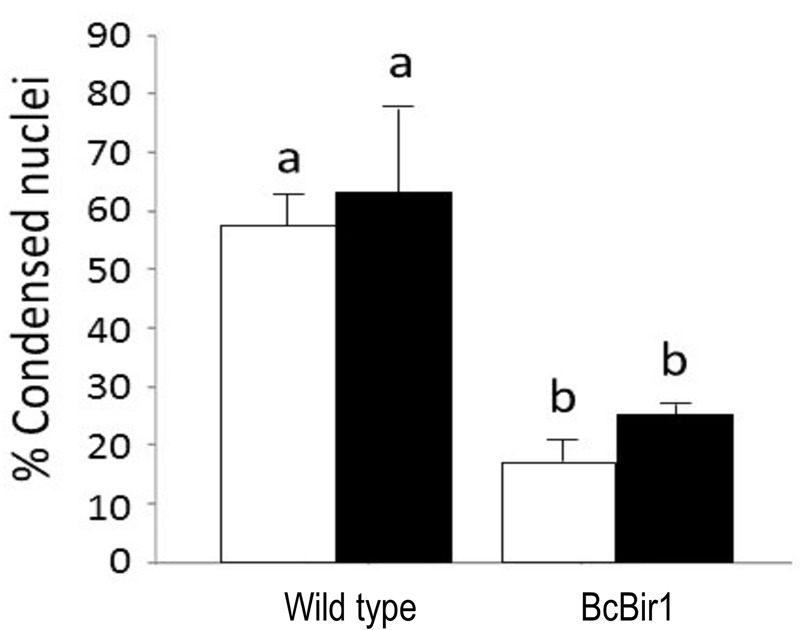
FIGURE 4: Quantification of chromatin condensation in hyphae of *B. cinerea *wild type and *BcBIR1* strains. Fungi were grown for 24 h in PDB medium at 22°C after which H_2_O_2_ was added to a final concentration of 10 mM. The cultures were incubated for additional 4 h, samples were removed, stained with Hoechst 33342 and then visualized at 1000x magnification. The percent of nuclei with condensed chromatin was scored and calculated manually (white bars), or by the Condensation module of the SCAN**^©^** software (black bars). Data are the mean average ± SEM of three samples, each containing at least 200 nuclei (n = 3). Percentage of apoptotic nuclei in untreated cultures was lower than 15% (not shown). Columns and lines not connected with the same letter are statistically different according to two-way ANOVA (P < 0.05) followed by a post-hoc Tukey HSD analysis (P < 0.001).

The Condensation module performs unbiased, automated quantification of condensed nuclei following staining with Hoechst 33342 or DAPI, and provides the ratio of condensed nuclei in a given population of hyphae. The parameters we used to discriminate intact nuclei from condensed nuclei included presence or absence of the nucleolus signal, distribution of the fluorescence within the nucleus, nuclei size, and relative fluorescence levels per nuclear area (see Materials and Methods for details). Using these parameters, we obtained accurate measurements of percent of condensed nuclei in samples of H_2_O_2_-treated and untreated hyphae (Fig. 4). The software results were slightly higher (but statistically not different) than the results obtained by manual counting. We attribute these differences to loss of information due to the strict criteria used in manual counts, which are applied to avoid user bias. Similar to the nuclei counting module, batch files can be automatically scored, a function that greatly facilitates the process.

**Figure 5:**
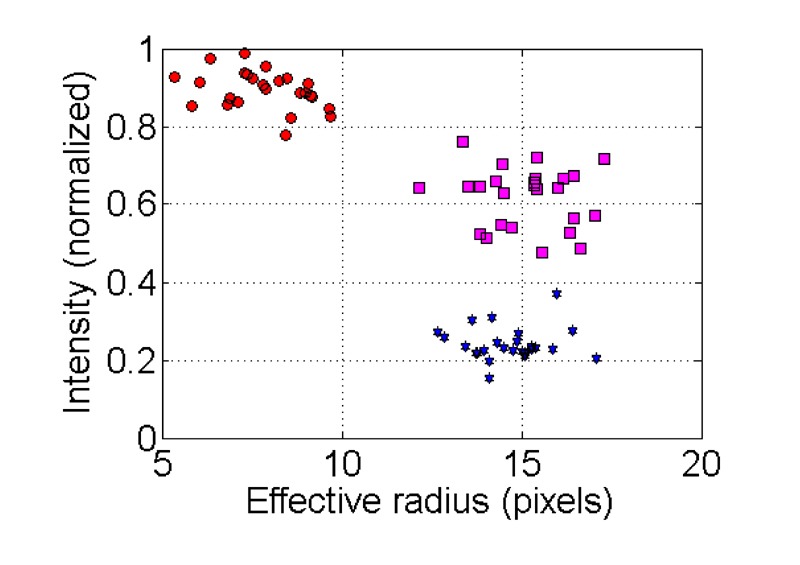
FIGURE 5: Illustration of nuclei distribution according to intensity and effective radius. Nuclei will affiliate to apoptotic, non-apoptotic, or intermediate states according to the values of their effective radius and intensity parameters. **Red circles** correspond to apoptotic nuclei (high intensity and low effective radius area); **blue stars** correspond to non-apoptotic nuclei data (low intensity and high effective radius area); **magenta squares** correspond to intermediate states (higher intensity and similar effective radius area compared with non-apoptotic nuclei).

The results obtained by the condensation module provided a good solution for scoring differences between relaxed and condensed nuclei. The differences in nuclei appearances could theoretically be used also to differentiate an intermediate state from non-apoptotic and from fully apoptotic cells, namely cells that undergo apoptosis, which are characterized by fragmented nuclei from cells at the end of the process, which are characterized by fully condensed nuclei (Fig. 3). Indeed, the output files include a third group, which is separate from the apoptotic and non-apoptotic groups (Fig. 5). This group is characterized by intermediate levels of fluorescence/nucleus, which fit with expected fluorescence in nuclei at intermediate stage of apoptosis. Surprisingly, the ratio of such intermediate nuclei remained unchanged in treated and untreated hyphae, namely there was no correlation between apoptosis levels as determined by ratio of condensed nuclei and the percent of intermediate nuclei. Thus, although a new population of nuclei was revealed by the software, we could not use it to improve assessments of the apoptotic stage of the cells. It might be that the dynamics of this population are such that there is a steady state level of intermediate cells, or that the software is not sensitive enough to distinguish between noisy samples and true intermediate nuclei. If the latter, then better images might improve the analysis and reveal changes in the levels of nuclei belonging to this population.

### The TUNEL module for counting of nuclei with DNA strand breaks

Cleavage of DNA by specific nucleases during apoptosis generates DNA fragments with defined sizes, which form a “ladder” when separated by gel electrophoresis. While DNA laddering is a hallmark of apoptosis in many organisms, it is hard to detect in fungi 28,29,9. Instead, end labeling of the DNA fragments using TUNEL is commonly used to detect DNA strand breaks in fungi 30,31,32,33. In the single-celled organisms it is possible to score TUNEL-positive nuclei by FACS, but in multicellular samples, such as fungal hyphae, the use of FACS is not possible. The way to obtain quantitative data in filamentous fungi is by manual counting of TUNEL-stained nuclei from a series of microscopic images, a tedious and difficult task.

**Figure 6:**
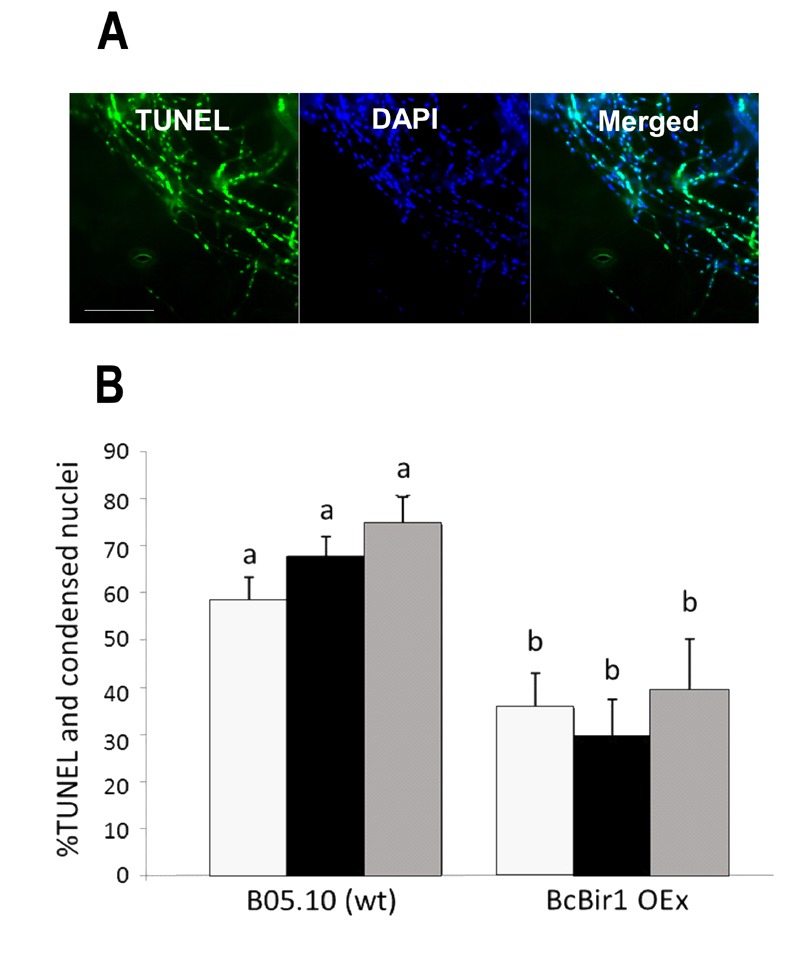
FIGURE 6: Quantification of TUNEL and comparison with chromatin condensation. **(A)** Images of infected leaves stained with TUNEL and DAPI. Leaves of *A. thaliana *cv. Col-0 were inoculated with conidia of *B. cinerea* wild type strain. TUNEL assay and DAPI staining were performed 48 h post inoculation, when massive PCD occurs in the fungus. TUNEL positive nuclei are detected in the GFP channel (left), DAPI-stained nuclei (all nuclei, including TUNEL positive and negative) are detected in the DAPI channel (middle). Yellow signal in the merge image indicates double stained nuclei (right). Bar = 20 µm. **(B)** A bar graph showing levels of TUNEL and DAPI stained nuclei. Cultures of wild type and a BcBir1 over expression (less sensitive to apoptosis) strains were produced in PDB at 22°C with agitation. After 24 h H_2_O_2_ was added to a final concentration of 10 mM and the cultures were incubated under the same conditions for additional 4 h. Mycelia were harvested, samples were processed for the TUNEL assay, and then stained with DAPI before microscopic visualization at 1000x magnification. The percent of TUNEL-positive nuclei was estimated manually **(black bars)** and by the TUNEL module of the software **(grey bars)**. Percent of nuclei with condensed DNA was determined in the same samples using the Condensation module of the SCAN**^©^** software **(white bars)**. Percentage of condensed and TUNEL-positive nuclei in untreated cultures was below 15% and 0.5%, respectively (not shown). All data are the mean average ± SEM of triplicate samples, each containing at least 200 nuclei per sample (n = 3). Columns and lines not connected with the same letter are statistically different according to two-way ANOVA (P < 0.05) followed by a post-hoc Tukey HSD analysis (P < 0.001).

To facilitate quantification of TUNEL-stained nuclei in hyphae, we developed a module of the SCAN**^©^** software that detects the nuclear signals in the DAPI and GFP channels (total and TUNEL-positive nuclei, respectively), and then calculates the ratio of TUNEL-stained nuclei. Similar results were obtained by manual scoring and SCAN**^©^** analysis (Fig. 6). Slightly reduced (statistically insignificant) levels of TUNEL-positive nuclei were obtained with the software, which are attributed to the rigid criteria that are used to define an apoptotic state. Because same criteria are used in all cases, there is no effect on the quality of the results.

Because nuclei were also stained with DAPI, we could measure DNA condensation in the same specimens and compare with the TUNEL results. The two parameters, as measured by the Condensation and the TUNEL modules, showed the same trend, namely high levels in wild type and reduced levels in the A-PCD-hyposensitive BcBir1 overexpression strains (Fig. 6B). Furthermore, the DNA condensation values were slightly higher than TUNEL values, which is in agreement with the earlier appearance of this marker 34. These results show that double staining and then automatic measurement of TUNEL- and DAPI-stained nuclei provide a reliable measure of apoptotic levels. Further, the strong correlation between results of DNA condensation and TUNEL suggests that DNA condensation may be used as a sole measure to estimate apoptosis. This is especially appealing since TUNEL is a rather difficult and expensive assay and therefore it can be applied simultaneously to a relatively small number of samples, whereas DAPI or Hoechst 33342 protocols are simple, highly reproducible, and can be performed simultaneously on a large number of samples.

### Concluding remarks

The SCAN**^©^** software enables automated quantification of stained nuclei and differentiation between nuclei in relaxed state to nuclei with condensed DNA or with DNA strand breaks. The results obtained by SCAN**^©^** analyses are comparable with results obtained by manual counting, the analyses can be performed on numerous samples and the results are validated statistically. As such, the SCAN**^©^** software provides a simple and easy solution for measurement of A-PCD in fungal hyphae as well as in other types of multicellular samples. It should be noted that we observed differences in results between live samples that were stained with Hoechst 33342 and samples after fixation that were stained either with Hoechst 33342 or with DAPI. For mycelia that were produced in liquid medium, we obtained reliable results only in live stained samples but not in samples after fixation (data not shown). In hyphae that were produced on a solid substance such as leaf surface, reliable results were obtained by staining samples after fixation (Fig. 6A). Comparison of Hoechst 33342-stained samples with or without fixation showed that the reason for the inaccurate results in samples after fixation was loss of the condensed chromosomal structure within the nucleus (Fig. S2). We are unsure why nuclei in hyphae that were produced on leaves were unaffected by the fixation. A possible explanation is that on leaves, the cytoskeleton is more rigid and hence the structure of nuclei is retained also after fixation.

## MATERIALS AND METHODS

### Fungal strains and growth conditions

*B. cinerea* Pers. [teleomorph *Botryotinia fuckeliana* (de Bary) Whetzel] wild type B05.10 and derived transgenic strains were used throughout this study: i) *BcBIR1, *a transgenic strain that overexpress the *BcBIR1 *anti-apoptotic gene and has reduced sensitivity to apoptotic-inducing conditions 34, ii) CA-BcRAC, a transgenic strain that expresses a constitutively active allele of the *BcRAC *gene and is hypernucleated 35. All strains were routinely cultured on potato dextrose agar (PDA). Strains were maintained at 21°C under continuous fluorescent light. For nuclear counting experiments, conidia of B05.10 and CA-BcRAC strains were collected from 7-day old cultures that were produced on PDA plates. Conidia were suspended in Gamborg’s B5 medium (Duchefa, The Netherlands) and the number of conidia was adjusted to 10^5^ conidia/ml. Samples of 50 µl were applied onto a glass slide and incubated for 24 h at 21°C in a humid chamber to allow germination and initial growth of the conidia.

For DNA condensation, hyphae from liquid cultures were stained with Hoechst 33342 (Sigma) without fixation. Hyphae on leaves were stained with DAPI (Sigma) following fixation with 3.7% formaldehyde 36. To test changes in DNA condensation following induction of A-PCD, conidia were suspended in potato dextrose broth (PDB, Acumedia) in 24-wells plates and incubated for 24 h at 21°C with agitation at 180 rpm. H_2_O_2_ was added to a final concentration of 10 mM and the cultures were incubated for additional 4 h. Samples of hyphae were removed after 4 h incubation with H_2_O_2_ and stained with Hoechst 33342.

### Staining procedures and microscopy

Nuclei were stained with Hoechst 33342 or with DAPI as previously described 34. Samples were visualized by a fluorescent microscope using a DAPI filter. DNA strand breaks were detected by TUNEL using the *In Situ* Cell Death Detection kit (Roche Applied Science, Indianapolis, IN), as previously described 37. Briefly, infected leaves were incubated in 3.7% formaldehyde for 40 min, then rinsed and digested with lysing enzyme (Sigma). Following treatment with the enzymes the tissue was rinsed twice with PBS and then incubated with 50 µl TUNEL reaction mix for 70 min at 37°C with gentle agitation. Stained samples were rinsed 3 times with PBS and then examined with a fluorescent microscope using the GFP filter. The percent of condensed and TUNEL positive nuclei was calculated by counting 200 nuclei per sample.

Epifluorescence and light microscopy were performed with a Zeiss Axio imager M1 microscope. Differential interference microscopy (DIC) was used for bright field images. The following filters were used for examination of fluorescent samples: DAPI filter (excitation 340 - 390 nm, emission 420 - 470 nm), GFP filter (excitation 450 - 490 nm, emission 500 - 550 nm). Images were captured at 400x or 1000x magnification with a Zeiss AxioCam MRm camera.

### The SCAN^©^ software

The SCAN**^©^** (System for Counting and Analysis of Nuclei) software was developed in MATLAB using algorithms realizations from the image processing toolbox, which were modified for our purposes. The software includes separate GUI for each module (see Supplementary Material, Fig. S1), which allows the user to define the relevant parameters and test the outcome, choose specific region(s) of interest, and perform analyses either on single or a set (library) of images. In all modules (Layers, Condensation, TUNEL), the images are processed in a single color plane, extracted from red, green and blue (RGB) color space representation. Each image analysis starts with segmentation of nuclei in the images by circles detection using Circle Hough Transform (CHT) method 38,39. The nuclei shapes are close enough to a circle to allow this segmentation approach. The CHT method was chosen due to its robustness in the presence of noise and sensitivity for finding local maxima. For optimization of the nuclei detection, the user can provide a range of possible radius values (in pixels) for the nuclei sizes and two additional threshold parameters that can be adjusted for edge gradient and sensitivity. The edge gradient parameter determines the edge pixels of the image, and the sensitivity parameter determines a circle in the accumulator space constructed by CHT. Remarkably, we found that for almost all experiments, a single set of parameters is sufficient, provided that all images are acquired at the same magnification.

#### Layers

For the Layers module, an algorithm was developed for distinguishing and counting nuclei in Hoechst 33342 or DAPI labeled images that were taken in a series of different focal planes (z-stacks). In each layer nuclei are detected as circles and a sharpness rating is determined for each circle. The circle that is created by most sharp edges that point to the center of the circle receives the highest score. This rating parameter is obtained directly from the CHT algorithm and represents the number of pointers to the circle in Hough parametric space. Circles in each layer are then compared to circles in adjacent layers and only the circles with the higher sharpness score are counted. This method ensures that each nucleus detection is matched to a specific layer.

#### Condensation

For the Condensation module, a method for detection and assessment of the percent of apoptotic nuclei was developed. The spatial distribution and intensity of the fluorescent signal in each nucleus is analyzed to calculate two parameters: an effective radius and intensity (normalized intensity per unit area). The nuclei will belong to apoptotic, non-apoptotic or intermediate states by the values of these parameters. The ranges, in the intensity-effective radius space that defines each state are pre-defined by the user as a calibration procedure for the software.

The data processing for calculating the intensity and effective radius is performed as follows: first, the pixels inside each nucleus circle are grouped into different intensity clusters (k = 4) using K-means algorithm 40,41. If the brightest cluster area occupies less than 15% of the total area of the circle and there is at least 20% brightness difference between the brightest and darkest clusters, the brightest cluster is identified as the nucleolus. The nucleolus is visible only in non-apoptotic nuclei (Fig. 3), and its intensity is removed from the nucleus intensity calculations to increase the distinction between apoptotic and non-apoptotic data. Accordingly, non-apoptotic nuclei are defined by two parameters: presence of a nucleolus (> 15% brightest area), which is removed from the total fluorescence count, and a difference in brightness between the brightest and darkest clusters that is lower than 20%. When the brightest cluster area is larger than 15% of the total area of the circle, and the difference between the brightest and darkest clusters is greater than 30%, the nucleus is defined as potentially apoptotic. In this case, the effective radius and fluorescence intensity are defined by this cluster data. In certain cases, such as intermediate states or noisy images, neither of these conditions is met. In these cases, the effective radius and fluorescence intensity are calculated from the total data in the nucleus circle detection. The values (15%, 20% and 30%) were chosen after comparative analyses of cluster data in a large number of images of apoptotic and non-apoptotic nuclei.

**Figure 7:**
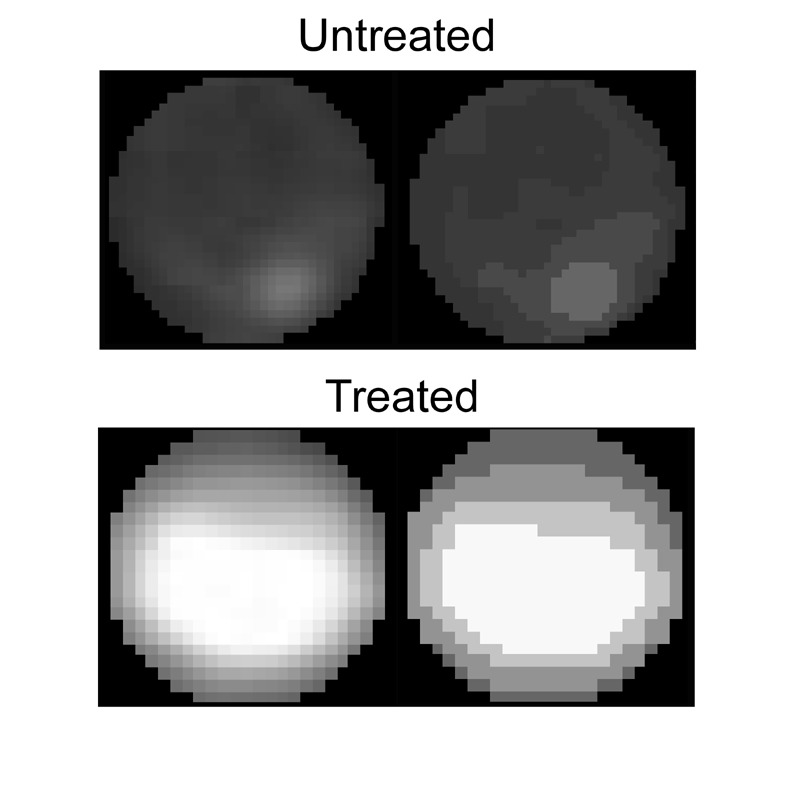
FIGURE 7: Data clustering. Non-apoptotic (top) and apoptotic (bottom) nuclei before and after the clustering to 4 brightness groups. For the calculation of the intensity and effective radius parameters: in the non-apoptotic nucleus the nucleolus pixels are not counted, in the apoptotic nucleus only the brightest cluster is counted.

Clustering of non-apoptotic and apoptotic nuclei is demonstrated in Figure 7. In the image of the non-apoptotic nucleus, the brightest cluster (the nucleolus) is removed and then the effective radius and intensity parameters are calculated. In the image showing an apoptotic nucleus, only the brightest cluster remains. In intensity vs. effective radius distribution plot, data points corresponding to apoptotic nuclei are grouped in the cluster of high intensity/low effective radius areas, (Fig. 5, red circles). Data points corresponding to non-apoptotic nuclei are grouped in the cluster of low intensity/high effective radius area (Fig. 5, blue stars). In high quality images (low noise levels) data points corresponding to intermediate state of nuclei can be distinguished. Nuclei in the intermediate category are grouped in a cluster that is characterized by higher fluorescence intensity than that of non-apoptotic nuclei but with a similar effective radius area (Fig. 5, magenta squares).

#### TUNEL 

In the TUNEL module, the ratio between the number of nuclei in GFP and DAPI channels is calculated. The TUNEL and DAPI labeled image pairs are aligned using an automatic intensity-based image registration with translation transformation 42. We expect to detect all the nuclei in the DAPI channel; hence, centers positions in the DAPI and GFP channels are compared for verification and in order to exclude false detections. Finally the nuclei are counted and the ratio is calculated.

#### Hardware

SCAN**^©^** software can run on a personal computer. At least 4GB memory is recommended for smooth and fast analyses. Images can be captured with all types of fluorescent microscopes. A 100x (1000 magnification), fluorescent objectives are recommended. It is also possible to use lower (63x or 40x) objectives if camera resolution is high enough.

#### Software availability

We will provide the software upon request and free of charge. To request the software please fill the agreement form provided in the supplementary material and send by email to the corresponding author (amirsh@ex.tau.ac.il).

## SUPPLEMENTAL MATERIAL

Click here for supplemental data file.

Agreement Form Click here for the agreement form.

All supplemental data for this article are also available online at http://microbialcell.com/researcharticles/measurement-of-apoptosis-by-scan-a-system-for-counting-and-analysis-of-fluorescently-labelled-nuclei/.
