# Supplementary material for: Measurement of apoptosis by SCAN©, a system for counting and analysis of fluorescently labelled nuclei
Source: Microb Cell. 2014 Nov 26;1(12):406–15. doi: 10.15698/mic2014.12.180 (PMC5349136; doi:10.15698/mic2014.12.180)
Supplement: Supplementary file 1 [file mic-01-406-s01.pdf]

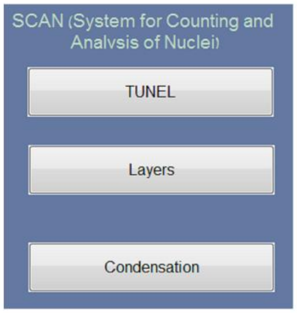

TUNEL

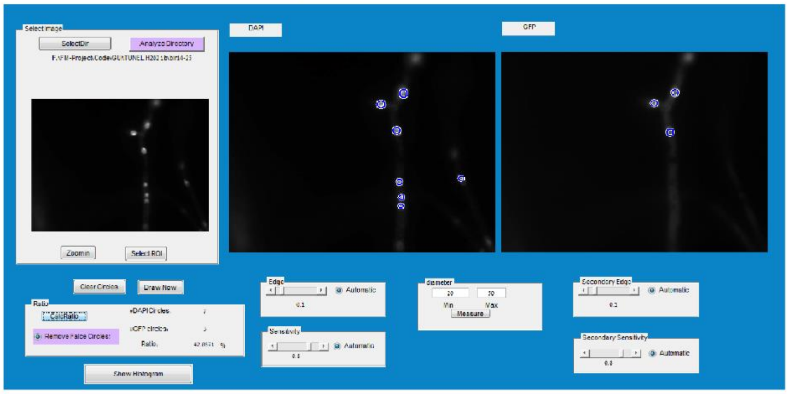

Layers

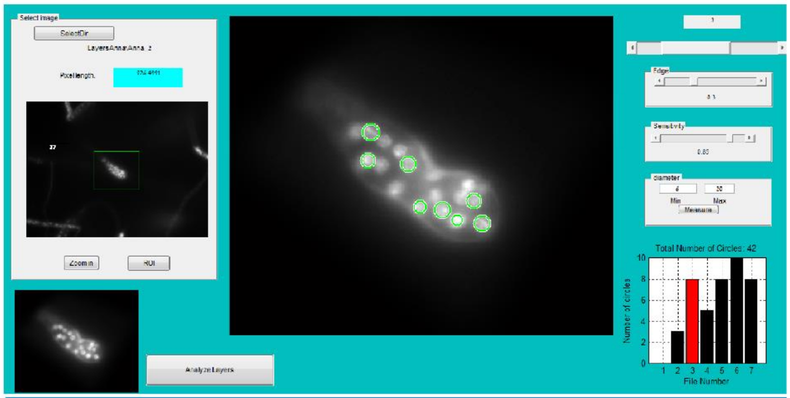

Condensation

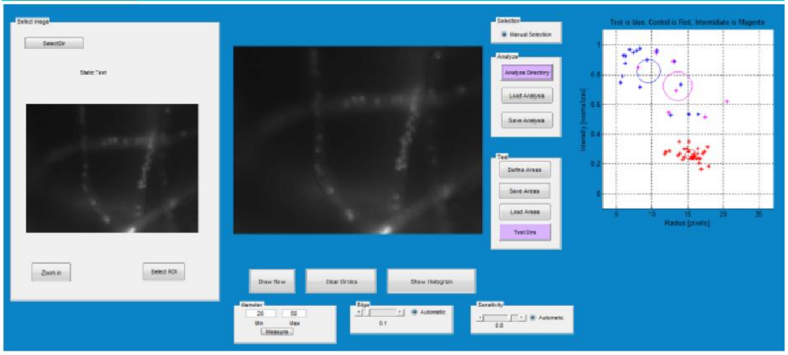

Figure S1. SCAN© software screenshots. In all modules there are boxes and bars to adjust the various parameters and a selection window (on the left) to define the region of interest in the image. Other buttons choose the library of images or run the analysis. **TUNEL** has two main windows, one for each channel (DAPI or GFP) where the nuclei detection are displayed. The ratio is shown on the bottom left of the screen. **Layers** has a main window that show the detection in a single layer, the layer can be chosen using the scroll bar on the right. The number of detected nuclei in each layer are displayed in the bottom right graph. **Condensation** has a main window that allows pre-run testing of the selected detection parameters. On the right, the distribution of the data is shown in intensity-effective radius graph. On that graph, the user can calibrate the software by marking the parameter ranges that define each state (apoptotic, non-apoptotic and intermediate)

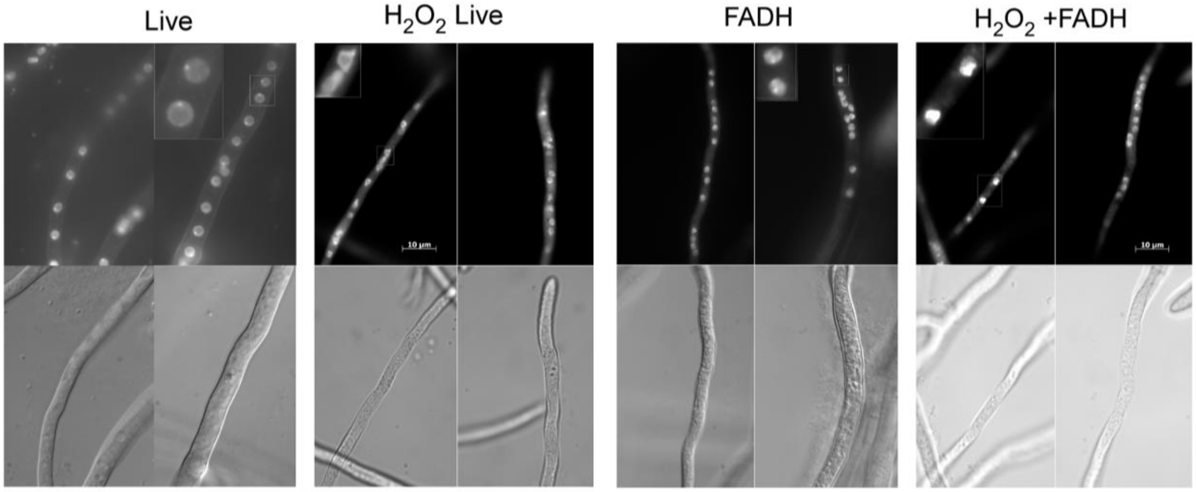

Figure S2. Differences in signal of Hoechst 33342-stained nuclei before and after fixation of the tissue with formaldehyde. Hyphae were produced in PDB for 24h and then incubated for additional 4 h in PDB with 10mM H_2O_2 (**H_2O_2**) or with an equal volume of water (**Control**). Samples were stained with Hoechst 33342 and then visualized by fluorescent microscopy without any further processing (**Live**) or following treatment with 3.7% formaldehyde (**FADH**).
