# Supplementary material for: Measurement of apoptosis by SCAN©, a system for counting and analysis of fluorescently labelled nuclei
Source: Microb Cell. 2014 Nov 26;1(12):406–15. doi: 10.15698/mic2014.12.180 (PMC5349136; doi:10.15698/mic2014.12.180)
Supplement: Agreement Form — Click here for the agreement form. [file mic-01-406-s02.pdf]

Request form for SCAN© software package

Personal detail:

Name: ____________________________________

Organization: ____________________________________

Address ____

____

____

Email: ___________________________________

Conditions:

1. SCAN© software will not be used for commercial purposes
2. SCAN© software will not be transferred to a third party without written permission from Prof. Amir Sharon
3. The manuscript by Shlezinger et al. 2014 will be cited in publications reporting results obtained with the aid of the SCAN© software
4. The authors have no liability and are not responsible in any way for problems that might arose from using the software

I have read and agree to these conditions:

Name ___________________ **Signature** _______________ Date _______________
